# Profiling of Potential Anti-Diabetic Active Compounds in White Tea: An Integrated Study of Polyphenol-Targeted Metabolomics, Network Pharmacology, and Computer Simulation

**DOI:** 10.3390/foods13213354

**Published:** 2024-10-22

**Authors:** Weiwei Wu, Zhiqiang Zheng, Zhihui Wang, Chenxi Gao, Yilin Liang, Wen Zeng, Weijiang Sun

**Affiliations:** College of Horticulture, Fujian Agriculture and Forestry University, Fuzhou 350002, China; wweiwei0601@163.com (W.W.); zzq000422@163.com (Z.Z.); wzh1246900265@163.com (Z.W.); gaochenxii@163.com (C.G.); lyl908213@163.com (Y.L.); wenzeng_t@163.com (W.Z.)

**Keywords:** white tea, diabetes, polyphenol-targeted metabolomics, network pharmacology, molecular dynamic simulation

## Abstract

Diabetes remains a critical global public health challenge, posing a growing threat to human health and well-being. White tea is a lightly fermented tea and one of the six traditional tea categories in China. Owing to its rich content of bioactive compounds such as catechins and alkaloids, it has demonstrated potential anti-diabetic properties. However, its precise bioactive components, mechanisms of action, and relevant molecular targets require further investigation. In this study, an integrated approach combining polyphenol-targeted metabolomics, in vitro antioxidant assays, *α*-glucosidase inhibition tests, network pharmacology analysis, GEO database exploration, molecular docking, and molecular dynamics simulations was employed to identify the potential anti-diabetic compounds, targets, and mechanisms of white tea. The findings revealed that white tea is particularly abundant in 10 bioactive compounds, including epigallocatechin gallate, epicatechin gallate, and catechin, all of which exhibit significant anti-diabetic potential. These compounds were found to exert their effects by interacting with core molecular targets, namely cathepsin V (CTSV) and nucleotide-binding oligomerization domain-containing protein 1 (NOD1), and engaging in pathways related to signal transduction, apoptosis, and immune responses. This study establishes a strong theoretical basis for advancing white tea research and underscores new opportunities for applying natural products in diabetes therapy.

## 1. Introduction

White tea (WT), one of the six traditional tea types in China, is produced from the tender leaves of *Camellia sinensis* and is renowned for its distinctive flavor and significant health benefits [1]. Primarily cultivated in Fujian Province, WT undergoes minimal processing, remaining unfermented, which preserves a high concentration of natural active compounds [2]. Research has indicated that WT is notably rich in catechins and alkaloids—compounds recognized for reducing oxidative stress and modulating immune responses—thus aiding in the prevention and management of chronic conditions such as cardiovascular disease, cancer, and diabetes [3].

Diabetes mellitus (DM) is a significant global public health issue, currently affecting over 500 million people, with projections suggesting that this number could rise to nearly 700 million by 2045 [4]. The pathogenesis of DM is closely associated with insulin resistance or insufficient insulin secretion, leading to abnormal elevations in blood glucose levels [5]. If left uncontrolled, DM can result in severe complications, including kidney and cardiovascular diseases, significantly increasing healthcare costs [6]. Reports indicate that individuals with diabetes utilize healthcare resources more frequently and extensively, leading to significantly higher medical expenses compared to those without diabetes [7]. Thus, developing effective strategies for preventing and treating DM is crucial for improving global public health.

In recent years, increasing attention has been given to the potential role of WT in the prevention and treatment of DM. Studies have shown that the polyphenol compounds in WT can effectively inhibit the activity of *α*-glucosidase [8]. Additionally, catechins alleviate DM-induced tubular injury by inhibiting endoplasmic reticulum stress and NLRP3-mediated inflammatory responses [9]. Furthermore, catechins exert their protective effects against DM by regulating the epithelial–mesenchymal transition (EMT) process through multiple pathways [10]. Despite these promising findings, the specific bioactive components, molecular mechanisms, and relevant targets of WT’s anti-DM effects require further elucidation.

Advances in metabolomics have significantly facilitated the identification and quantification of polyphenolic compounds, enabling deeper insights into their bioactivities [11]. Furthermore, developments in bioinformatics and related databases have promoted the application of network pharmacology in drug research, revealing the intricate relationships between bioactive compounds, targets, signaling pathways, and diseases [12]. Molecular docking analyses are frequently used to validate the interactions between bioactive compounds identified through network pharmacology and their target proteins, while molecular dynamics simulations further refine the binding modes of these compounds, offering a more accurate understanding of these interactions at the molecular level [13].

The present study aims to systematically investigate the anti-DM active components of WT through the integration of polyphenol-targeted metabolomics, in vitro antioxidant activity assays, *α*-glucosidase inhibition experiments, network pharmacology, GEO database analysis, molecular docking, and molecular dynamics simulations. By identifying the key molecular targets and elucidating the underlying mechanisms, this research provides a theoretical foundation for the comprehensive utilization of WT and offers new insights into the application of natural products in DM therapy.

## 2. Materials and Methods

### 2.1. Tea Samples and Chemicals

Tea leaf samples of the *Camellia sinensis* (L.) O. Kuntze cv. Fuding Dahao cultivar, specifically fresh leaves classified as one bud with one or two leaves, were sourced from Liumiao White Tea Co., Ltd., located in Fuding City, China. In the spring of 2024, fresh leaves at the one-bud-two-leaf stage were harvested and immediately transported to the withering facility. The leaves were evenly spread on bamboo trays, maintaining a thickness of 2–3 cm, and subjected to natural indoor withering for 48 h under controlled conditions (ambient temperature: 20–25 °C, humidity: 60–70%). Following the withering process, the leaves were transferred to a drying machine and dried at 60 °C for 3 h, producing the WT samples. After collection, all samples were stored in the dark at −20 °C until further analysis.

Vitamin C was obtained from Macklin Biochemical Technology Co., Ltd. (Shanghai, China), while acarbose was provided by Nanjing Dolay Biotechnology Co., Ltd. (Nanjing, China). Antioxidant assay kits for DPPH, ABTS, FRAP, and HRSA, as well as the *α*-glucosidase activity assay kits, were purchased from Suzhou Comin Biotechnology Co., Ltd. (Suzhou, China). Standards including gallic acid (GA, >99%), epigallocatechin gallate (EGCG, >99%), epicatechin (EC, >99%), catechin gallate (CG, >99%), epigallocatechin (EGC, >99%), catechin (C, >99%), gallocatechin gallate (GCG, >99%), epicatechin gallate (ECG, >99%), caffeine (>99%), theophylline (>99%), and theobromine (>99%) were purchased from Sigma-Aldrich (St. Louis, MO, USA). Methanol, acetonitrile, and acetic acid (HPLC grade) were sourced from Aladdin Biochemical Technology Co., Ltd. (Shanghai, China).

### 2.2. Determination of Antioxidant Activity and α-Glucosidase Inhibition of WT

The antioxidant activities of WT, including the 2,2-diphenyl-1-picrylhydrazyl (DPPH) radical scavenging assay, the 2,2′-azino-bis(3-ethylbenzothiazoline-6-sulfonic acid) (ABTS) radical scavenging assay, the ferric reducing antioxidant power (FRAP) assay, and the hydroxyl radical scavenging activity (HRSA) assay, as well as its inhibitory effect on α-glucosidase activity, were determined using respective assay kits. Vitamin C was used as the positive control in the antioxidant assays, while distilled water was the negative control. For the *α*-glucosidase inhibition assay, acarbose was used as the positive control and distilled water as the negative control.

#### 2.2.1. DPPH Radical Scavenging Activity

To determine the DPPH radical scavenging activity, the method described by Peng et al. was followed [14]. A 0.1 g of tea powder or vitamin C was mixed with 1 mL of extraction solution 1, and the mixture was homogenized on ice. The homogenate was centrifuged at 10,000× *g* for 10 min at 4 °C. The supernatant was collected and stored on ice for analysis. A 20 μL aliquot of each sample (WT, vitamin C, or distilled water) was mixed with 380 μL of reaction solution 1 (A_test1_) and incubated in the dark at room temperature for 20 min. After incubation, 200 μL of the mixture was transferred to a 96-well plate for absorbance measurement at 515 nm. A blank control (A_blank1_) was prepared by adding 20 μL of extraction solution to 380 μL of reaction solution. The DPPH radical scavenging activity was calculated as follows:

(1) $$DPPH\left(\%\right)=\frac{A_{blank1}-A_{test1}}{A_{blank1}}\times 100\%,$$

#### 2.2.2. ABTS Radical Scavenging Activity

For the ABTS assay, the method described by Yan et al. was followed [15]. A mixture of 0.1 g of tea powder or vitamin C and 1 mL of extraction solution 2 was prepared and homogenized on ice. After centrifugation at 10,000× *g* for 10 min at 4 °C, the supernatant was collected and kept on ice. A 10 μL aliquot of each sample (WT, vitamin C, or distilled water) was mixed with 190 μL of reaction solution 2 (A_test2_) and incubated for 10 min. The absorbance at 734 nm was then measured. A blank control (A_blank2_) was prepared by mixing 10 μL of extraction solution with 190 μL of reaction solution. The ABTS radical scavenging activity was calculated as follows:

(2) $$ABTS\left(\%\right)=\frac{A_{blank2}-A_{test2}}{A_{blank2}}\times 100\%,$$

#### 2.2.3. HRSA Radical Scavenging Activity

To evaluate HRSA, the method described by Chen et al. was employed [16]. A mixture of 0.1 g of tea powder or vitamin C and 1 mL of extraction solution 3 was homogenized on ice, followed by centrifugation at 10,000× *g* for 10 min at 4 °C. The supernatant was placed on ice for subsequent analysis. A 200 μL aliquot of each sample (WT, vitamin C, or distilled water) was mixed with 100 μL of reaction solution 3, 100 μL of reaction solution 4, 200 μL of reaction solution 5, and 300 μL of distilled water (A_test3_), and incubated at 37 °C for 20 min. A 200 μL aliquot of the reaction mixture was transferred to a 96-well plate, and the absorbance was measured at 510 nm. In the control group (A_control1_), 100 μL of reaction solution 3, 100 μL of reaction solution 4, 200 μL of reaction solution 5, and 500 μL of distilled water were used, while in the blank group (A_blank3_), 100 μL of reaction solution 3, 200 μL of reaction solution 5, and 600 μL of distilled water were used. The HRSA was calculated using the following formula:

(3) $$HRSA\left(\%\right)=\frac{A_{blank2}-A_{test3}}{A_{blank3}}\times 100\%,$$

#### 2.2.4. FRAP Radical Scavenging Activity

For the FRAP assay, the method described by Jiao et al. was followed [17]. A mixture of 0.1 g of tea powder or vitamin C and 1 mL of extraction solution 4 was homogenized on ice and then centrifuged at 10,000× *g* for 10 min at 4 °C. The supernatant was collected and placed on ice for further analysis. A 10 μL aliquot of each sample (WT, vitamin C, or distilled water) was mixed with 190 μL of reaction solution 6 (A_test4_), and the mixture was incubated for 20 min. The absorbance at 593 nm was measured in a 96-well plate. A blank control (A_blank4_) was prepared by adding 10 μL of extraction solution to 190 μL of reaction solution. The FRAP value (μmol Trolox/g) was calculated as follows:

(4) $$FRAP\left(\mu mol\;Trolox/g\right)=\frac{0.8054\times [{(A}_{test4}-A_{blank4})-0.0134]}{0.1},$$

#### 2.2.5. α-Glucosidase Inhibition Activity

To determine the inhibitory effect of WT on α-glucosidase activity, the method described by Yu et al. was followed [18]. A mixture of 0.1 g of tea powder or acarbose and 1 mL of extraction solution 5 was homogenized on ice, followed by centrifugation at 15,000× *g* for 10 min at 4 °C. The supernatant was collected and stored on ice for analysis. A 30 μL aliquot of each sample (WT, acarbose, or distilled water) was mixed with 120 μL of reaction solution 7 and 150 μL of reaction solution 8 (A_test5_) and incubated at 37 °C for 30 min. The reaction mixture was then transferred to a 95 °C water bath for 5 min. After cooling, the mixture was centrifuged at 8000× *g* for 5 min at 4 °C. A 70 μL aliquot of the supernatant was mixed with 130 μL of reaction solution 9, and the mixture was incubated at room temperature for 2 min before measuring absorbance at 400 nm. In the control group (A_control2_), 30 μL of sample (WT, acarbose, or distilled water) was mixed with 120 μL of distilled water and 150 μL of reaction solution 8. The *α*-glucosidase inhibitory activity was calculated using the following formula:

(5) $$\alpha -Glucosidase\;inhibition(\%)=\frac{{85.47\times [(A}_{test5}-A_{control2})]}{0.1}$$

### 2.3. Targeted Polyphenol Metabolomics Analysis

Alkaloids (theophylline, theobromine, caffeine), gallic acid (GA), and catechins (GC, EGC, C, EC, EGCG, GCG, ECG) were quantified using a high-performance liquid chromatography (HPLC) system (E2695, Waters, Milford, MA, USA) equipped with a Phenyl-Hexyl 100A column (250 mm × 4.6 mm, 5 μm, Phenomenex, Los Angeles, CA, USA). Sample preparation was performed according to the Chinese National Standard GB/T 8313—2018, which specifies methods for the determination of polyphenols and catechins in tea [19]. The mobile phase consisted of solution A (2% acetic acid) and solution B (100% acetonitrile). The gradient conditions were set as follows: 0–25 min (91% A, 9% B); 25–35 min (68.3% A, 31.7% B); 35–40 min (100% B). The flow rate was 1 mL/min, with an injection volume of 10 μL. The column temperature was maintained at 35 °C, and the detection wavelength was set at 278 nm for ultraviolet (UV) detection.

### 2.4. Screening of WT Active Components’ Targets and Construction of WT-Component-Target Network

The SMILES information for alkaloids (theophylline, theobromine, caffeine), gallic acid (GA), and catechins (GC, EGC, C, EGCG, GCG, and ECG) was retrieved from the PubChem database (https://pubchem.ncbi.nlm.nih.gov/, accessed on 10 July 2024). The potential targets of these compounds were predicted using the SwissTargetPrediction (http://www.swisstargetprediction.ch/, accessed on 10 July 2024) and SEA (https://sea.bkslab.org/, accessed on 10 July 2024) databases. Subsequently, a network of active components and their target genes was constructed using Cytoscape (version 3.9.1), revealing the core relationships between WT components and their targets.

### 2.5. Identification of Differentially Expressed Genes (DEGs) in DM

Gene expression profiles related to DM were obtained from human samples in the GEO database (http://www.ncbi.nlm.nih.gov/geo/, accessed on 10 July 2024). Differential gene analysis was conducted using the R package “limma”, and results were displayed in boxplots based on the criteria of *p* < 0.05 and |log_2_FC| > 0.5 (Supplementary Figure S1A,B). A volcano plot was generated using the “ggplot2” package in R (version 4.3).

### 2.6. Weighted Gene Co-Expression Network Analysis (WGCNA)

Gene modules were defined using the WGCNA algorithm, which identifies groups of genes with similar expression patterns. The weighted gene co-expression network was constructed using the “WGCNA” package in R. Soft threshold power was chosen based on the criterion of scale-free topology (R^2^ > 0.85). The adjacency matrix was transformed into a topological overlap matrix (TOM) to quantify the degree of overlap between genes. Modules were identified using hierarchical clustering and dynamic tree-cut methods, with a minimum module size of 30 genes. Modules showing a high degree of similarity (>0.75) were merged to reduce redundancy, and module-trait relationships were calculated using Pearson correlation coefficients. Different modules were represented by distinct colors, and modules with similar expression patterns were merged. The modules with the highest correlations to healthy and DM samples were selected, and genes closely associated with these samples were extracted for further analysis.

### 2.7. Screening of Key Targets and Functional Enrichment Analysis

Potential anti-DM therapeutic targets were identified by intersecting the predicted targets of WT active components, DEGs, and genes from the pink and turquoise modules using the “UpSetR” package in R. To explore the biological functions of the identified key target genes, Gene Ontology (GO) analysis and KEGG pathway enrichment were performed using the “clusterProfiler” package, and the results were ranked by *p*-value. GO analysis included terms from three categories: molecular function (MF), cellular component (CC), and biological process (BP). The top 10 most significant terms (*p* < 0.05) from each category were selected and visualized using the “GOplot” package. Similarly, the top 10 most significant KEGG pathways (*p* < 0.05) were visualized with the “GOplot” package

### 2.8. Molecular Docking Verification

The diagnostic performance of key targets in AD was evaluated using receiver operating characteristic (ROC) curves, and the area under the curve (AUC) was calculated using the “pROC” package. Targets with an AUC greater than 0.7 were identified as core targets. The expression differences in core targets between healthy and AD samples were analyzed using boxplots and visualized with the “ggplot2” package.

Ligand files in SDF format for 10 WT active components, including EGCG and ECG, as well as the positive drug acarbose, were obtained from the PubChem database. The 3D structure models of the CTSV (PDB ID: 7PK4) and NOD1 (PDB ID: 4JQW) proteins were retrieved from the UniProt database (https://www.uniprot.org/, accessed on 10 July 2024). Molecular docking was performed using AutoDock Vina 1.2.5 with an exhaustiveness value of 8. The docking results were evaluated based on the binding affinity (expressed in kcal/mol), and poses with root mean square deviation (RMSD) values of less than 2 Å were considered valid. The docking results were further analyzed and visualized with Discovery Studio 2019.

### 2.9. Molecular Dynamics Simulation

To further investigate the stability of protein-ligand interactions, molecular dynamics (MD) simulations of the EGCG-CTSV, ECG-CTSV, and C-NOD1 complexes were performed using GROMACS 2020.6 software. The AMBER99SB force field and SPC water model were applied, with a time step of 2 fs, and the particle mesh Ewald (PME) method was used for long-range electrostatic interactions. The system was equilibrated for 100 ps under NVT conditions and 100 ps under NPT conditions. The production MD run was carried out for 50 ns at 300 K with a cut-off distance of 1.2 nm. The root mean square deviation (RMSD), root mean square fluctuation (RMSF), and potential energy were monitored to confirm system stability and convergence. Energy minimization was performed first, followed by equilibration to stabilize the system, after which MD simulations were conducted. Free energy calculations were carried out using MM/GBSA and MM/PBSA methods, and the results were visualized using Xmgrace 5.1.25 software.

### 2.10. Statistical Analysis

Statistical analysis was conducted using SPSS 19.0, and all data were presented as mean ± standard deviation (SD). One-way analysis of variance (ANOVA) was used, followed by post hoc analysis with Duncan’s multiple range test. A value of *p* < 0.05 was considered statistically significant.

## 3. Results and Discussion

### 3.1. Antioxidant Activity and α-Glucosidase Inhibition of WT

Antioxidants play a critical role in protecting pancreatic *β*-cell function by alleviating oxidative stress. This protection contributes to the management of DM and its complications [20]. *α*-Glucosidase inhibitors are crucial in controlling postprandial blood glucose levels by delaying carbohydrate absorption [21]. Therefore, the antioxidant activity and *α*-glucosidase inhibitory activity of WT were evaluated in this study. The results demonstrated that the DPPH, ABTS, HRSA, and FRAP values in the Control group were 1.31%, 1.27%, 0.64%, and 0.13 μmol Trolox/g, respectively, while in the WT group, these values were 71.50%, 85.43%, 6.57%, and 9.46 μmol Trolox/g, respectively. In the Vitamin C group, the DPPH, ABTS, HRSA, and FRAP values were 74.50%, 94.92%, 15.99%, and 28.49 μmol Trolox/g, respectively (Table 1). Statistical analysis indicated that both the WT and Vitamin C groups had significantly higher DPPH values compared to the Control group, and the Vitamin C group exhibited significantly higher ABTS, HRSA, and FRAP values than both the WT and Control groups. Additionally, the WT group showed significantly higher ABTS, HRSA, and FRAP values than the Control group. In the *α*-glucosidase inhibition assay, the *α*-glucosidase activity in the acarbose, WT, and Control groups was 8.74 nmol/min/g, 14.09 nmol/min/g, and 163.13 nmol/min/g, respectively. As shown in Table 1, *α*-glucosidase activity was significantly higher in the control group compared to both the acarbose and WT groups. The results of this study align with those reported by Zhou et al. and Esposito et al., confirming that WT demonstrates pronounced antioxidant activity and significant inhibition of α-glucosidase [8,22]. Collectively, these findings suggest that WT may offer promising antidiabetic properties.

### 3.2. Screening of Key Active Components and Relevant Targets of WT

**Figure 1:**
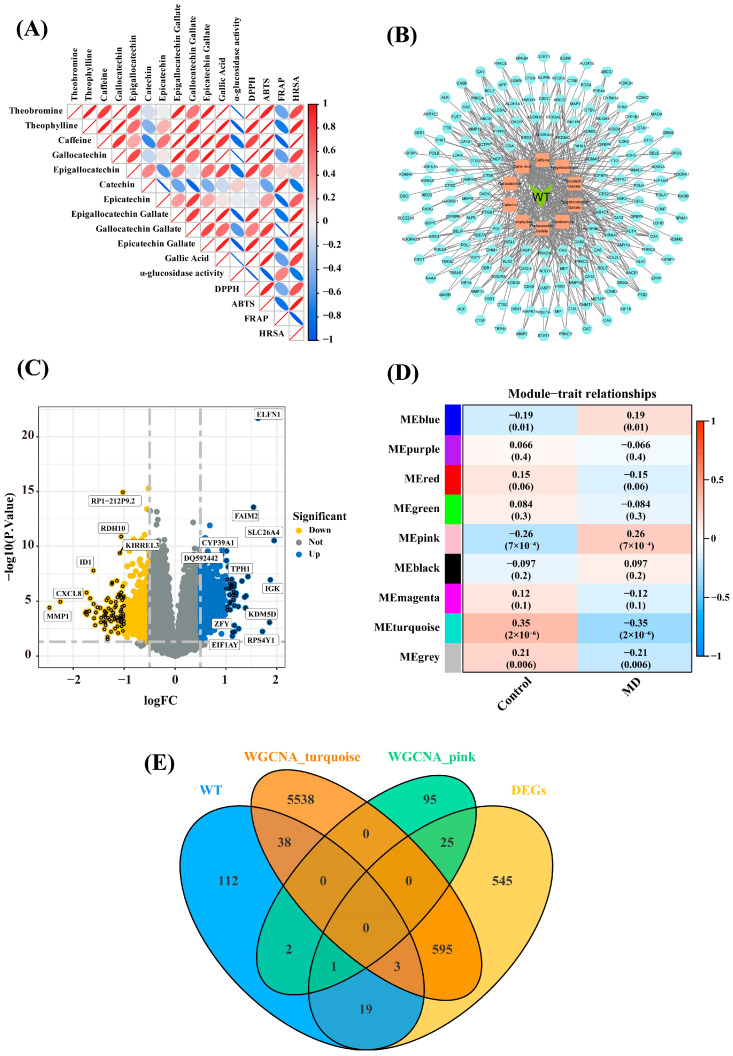
(**A**) The correlation between WT bioactive compounds and antioxidant activity and α-glucosidase inhibition indices. Correlation coefficients are displayed in a heatmap, where blue indicates a negative correlation, and red signifies a positive correlation; (**B**) A WT-bioactive compound-target network diagram. In the diagram, WT is shown in Yellow Green, bioactive compounds in Light Salmon, and their targets in Pale Turquoise. Each line connecting the compounds and targets represents potential interactions; (**C**) Volcano plot of differentially expressed genes (DEGs) from GSE76896. Blue dots represent upregulated genes, and yellow dots represent downregulated genes; (**D**) Heatmap showing correlations between gene co-expression modules and phenotypic traits. Red represents positive correlations, while blue represents negative correlations; (**E**) Venn diagram.

Targeted polyphenol metabolomics analysis (Figure 1, Supplementary Table S1) revealed that the contents of theophylline, theobromine, and caffeine were 1.24 mg/g, 1.52 mg/g, and 39.87 mg/g, respectively, while the content of GA was 0.73 mg/g. The contents of catechins, including GC, EGC, C, EC, EGCG, GCG, and ECG, were 5.97 mg/g, 7.07 mg/g, 0.75 mg/g, 5.24 mg/g, 102.63 mg/g, 7.25 mg/g, and 23.7 mg/g, respectively. To further identify potential anti-DM active components of WT, a correlation analysis was conducted between these components and antioxidant activity as well as *α*-glucosidase inhibitory activity. The results (Figure 1A, Supplementary Table S2) indicated significant positive correlations (r > 0.8) between DPPH and theophylline, theobromine, GC, EGC, EGCG, ECG, and GA. ABTS was significantly positively correlated (r > 0.8) with theophylline, theobromine, caffeine, GC, GA, EGCG, GCG, and ECG. HRSA showed significant positive correlations (r > 0.8) with theophylline, caffeine, EGCG, GCG, and ECG. Additionally, FRAP exhibited a significant positive correlation (r > 0.8) with C, while *α*-glucosidase inhibitory activity was significantly negatively correlated (r < −0.8) with theophylline, theobromine, caffeine, GC, EGCG, ECG, and GA. Based on these findings, theophylline, theobromine, caffeine, GA, GC, EGC, C, EGCG, GCG, and ECG were identified as potential anti-DM active components of WT, and these 10 components were subjected to further analysis. The SMILES information for these compounds was obtained from the PubChem database, and their potential targets were predicted using the SwissTargetPrediction and SEA databases. After removing duplicates, a total of 175 potential targets and their corresponding gene symbols were identified (Figure 1B, Supplementary Table S3). Theophylline was associated with 40 potential targets, theobromine with 42, caffeine with 48, GA with 72, GC with 13, EGC with 13, C with 24, EGCG with 56, GCG with 56, and ECG with 57 potential targets.

### 3.3. Screening of Key Genes in DM

In this study, the GSE76896 dataset was selected for analysis, which contains 171 pancreatic islet samples, including 116 from healthy individuals and 55 from DM patients (Supplementary Table S4). A total of 14,435 genes were identified from the GSE76896 dataset. DEGs were screened based on the criteria of *p* < 0.05 and |log2FC| > 0.5. By comparing healthy and DM samples, 1195 DEGs were identified, with 645 genes being upregulated and 550 downregulated (Figure 1C, Supplementary Table S5). WGCNA was used to construct a scale-free gene co-expression network, correlating gene expression patterns with clinical data. By correlating gene expression patterns with clinical data, this systems biology approach overcomes the limitations of traditional methods that focus solely on gene expression without considering gene-gene relationships [23]. WGCNA of the 14,435 genes resulted in the division of these genes into nine co-expression modules, and the correlation between these modules and both healthy and DM samples was displayed (Figure 1D). Among the identified modules, the pink and turquoise modules exhibited significant correlations with DM samples. The pink module (125 genes) was positively correlated with DM samples (r = −0.26, *p =* 7 × 10^−4^) and negatively correlated with healthy samples (r = −0.26, *p =* 7 × 10^−4^). Conversely, the turquoise module (6231 genes) was negatively correlated with DM samples (r = −0.35, *p =* 2 × 10^−6^) and positively correlated with healthy samples (r = 0.35, *p =* 2 × 10^−6^). Based on these findings, the clinical significance of the pink and turquoise modules was further explored.

To identify key anti-DM genes affected by WT components, an intersection analysis was conducted between the predicted targets of WT active components, DEGs, and genes from the pink and turquoise modules. Figure 1E shows the overlap between predicted targets of WT active components and DEGs. This analysis identified 19 intersecting genes. Additionally, two intersecting genes were found between WT targets and the pink module, while 38 were found between WT targets and the turquoise module. Additionally, one gene overlapped between the WT targets, DEGs, and the pink module, and three genes overlapped between the WT targets, DEGs, and the turquoise module (Supplementary Table S6). After deduplication, a total of 63 intersecting genes were identified, suggesting that WT active components may exert their anti-DM effects by targeting these 63 key genes.

### 3.4. Functional Enrichment Analysis of Key Targets

**Figure 2:**
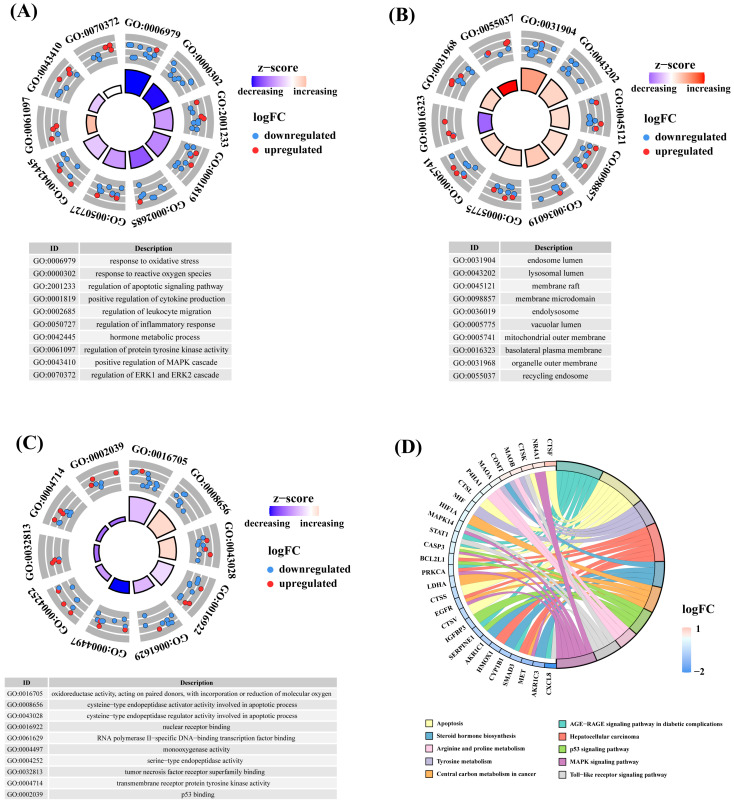
Functional enrichment analysis of key targets was performed as follows. (**A**) Biological process (BP) enrichment analysis of key targets; (**B**) Cellular component (CC) enrichment analysis of key targets; (**C**) Molecular function (MF) enrichment analysis of key targets; (**D**) KEGG pathway enrichment analysis of key targets.

To further elucidate the biological functions of the key targets, GO and KEGG enrichment analyses were performed on the 63 key genes. GO enrichment analysis showed that these genes were enriched in 548 BP terms, 23 CC terms, and 94 MF terms, accounting for 82.40%, 3.46%, and 14.14% of the total, respectively. In the BP category, the key targets were primarily enriched in processes related to signal transduction and apoptosis (Regulation of apoptotic signaling pathway, Positive regulation of MAPK cascade, Regulation of ERK1 and ERK2 cascade and Regulation of protein tyrosine kinase activity), immune and inflammatory responses (Positive regulation of cytokine production, Regulation of leukocyte migration, and Regulation of inflammatory response), responses to external stimuli and cellular stress (Response to oxidative stress and Response to reactive oxygen species), and Hormone metabolic process (Figure 2A). In the CC category, the key targets were mainly enriched in membrane structures and associated regions (Membrane raft, Membrane microdomain, Mitochondrial outer membrane, Basolateral plasma membrane, and Organelle outer membrane), endosome and lysosome-associated structures (Endosome lumen, Lysosomal lumen, Endolysosome, and Recycling endosome), and Vacuolar lumen (Figure 2B). In the MF category, the key targets were enriched in protease activity and regulation (Cysteine-type endopeptidase activator activity involved in apoptotic process, Cysteine-type endopeptidase regulator activity involved in apoptotic process and Serine-type endopeptidase activity), signal transduction and receptor binding (Tumor necrosis factor receptor superfamily binding and Transmembrane receptor protein tyrosine kinase activity), enzymatic activity and redox reactions (Oxidoreductase activity, acting on paired donors, with incorporation or reduction in molecular oxygen and Monooxygenase activity), and interactions with proteins and transcriptional regulators (Nuclear receptor binding, RNA polymerase II-specific DNA-binding transcription factor binding and p53 binding) (Figure 2C). Additionally, KEGG pathway analysis revealed that the key targets were primarily involved in signaling and disease-related pathways (AGE-RAGE signaling pathway in diabetic complications, p53 signaling pathway, Toll-like receptor signaling pathway, and MAPK signaling pathway), apoptosis and cancer-related pathways (Apoptosis, Hepatocellular carcinoma, Central carbon metabolism in cancer, and Lipid and atherosclerosis), and metabolic and biosynthesis pathways (Tyrosine metabolism, Steroid hormone biosynthesis, and Arginine and proline metabolism) (Figure 2D). These results from GO and KEGG pathway enrichment analyses suggest that the key targets play significant roles in signal transduction, apoptosis, and immune responses.

### 3.5. Screening of Core Targets

**Figure 3:**
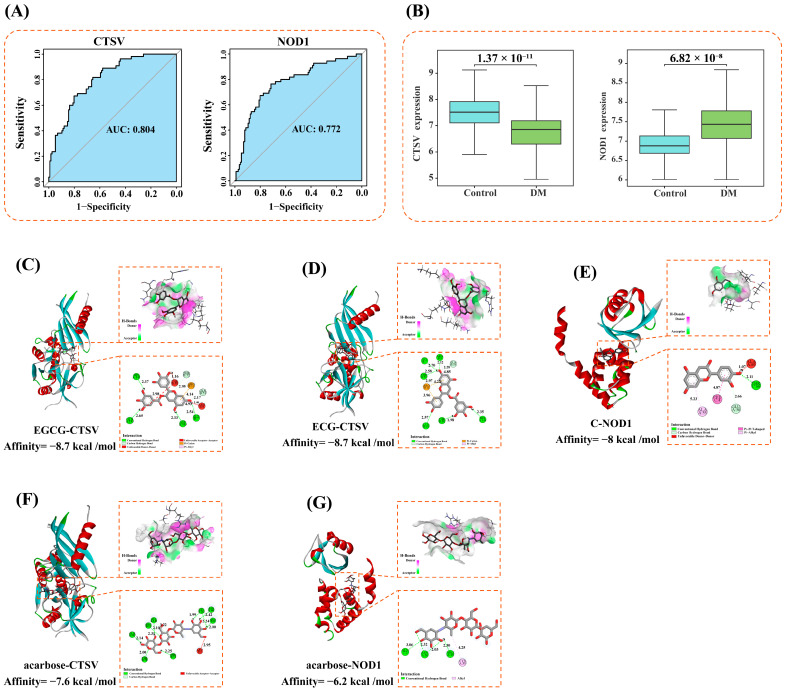
(**A**) Receiver Operating Characteristic (ROC) curve analysis of CTSV and NOD1; (**B**) Box plot comparing CTSV and NOD1 gene expression levels between the Control group (light blue) and DM group (light green), with error bars representing variability; (**C**) Interaction diagram of the EGCG-CTSV complex; (**D**) Interaction diagram of the ECG-CTSV complex; and (**E**) Interaction diagram of the C-NOD1 complex; (**F**) Interaction diagram of the acarbose-CTSV complex; (**G**) Interaction diagram of the acarbose—NOD1 complex.

The ROC curve is commonly used to evaluate the diagnostic efficiency of core genes, with an AUC greater than 0.7 typically indicating high diagnostic value [24]. In this study, 60 key genes were evaluated using ROC curves, with AUC > 0.75 set as the screening threshold. The results indicated that two targets, CTSV and NOD1, had AUC values exceeding 0.75 (Figure 3A). To verify the reliability of the ROC curve results, box plots were employed to analyze the differential expression of these core targets between healthy and DM samples. It was observed that NOD1 was significantly upregulated in DM samples, whereas CTSV was significantly downregulated (*p* < 0.05) (Figure 3B). NOD1, a receptor involved in immune responses, can trigger inflammation by activating the NF-κB and MAPK signaling pathways, which interfere with insulin signaling and lead to insulin resistance [25]. In adipose tissue, the activation of NOD1 increases lipolysis and reduces insulin-stimulated glucose uptake, further exacerbating glucose metabolism disorders [26]. The aberrant activation of NOD1 has been strongly linked to chronic low-grade inflammation, a critical pathological hallmark of type 2 diabetes [27]. Consequently, NOD1 is believed to play a pivotal role in the development and progression of DM. Therefore, therapeutic strategies targeting NOD1 could hold significant potential for the treatment of DM by mitigating inflammation-associated disease mechanisms. CTSV, a cysteine protease mainly active in lysosomes, is involved in protein degradation [28]. CTSV plays a critical role in apoptosis, with its dysregulated expression accelerating the apoptosis of pancreatic *β*-cells, a key contributor to type 2 diabetes pathogenesis [29]. Thus, targeting CTSV may protect pancreatic islet function by reducing *β*-cell apoptosis, offering a promising therapeutic approach for DM management. Understanding the precise function of these core targets in DM pathology holds significant clinical relevance. Firstly, these core targets could serve as diagnostic biomarkers, facilitating early detection and risk assessment of DM. Secondly, targeting these core genes and their related pathways may offer new avenues for the development of DM therapeutic strategies. Modulating their expression or activity could potentially aid in the regulation of immune responses and slow the progression of DM.

### 3.6. Molecular Docking

Molecular docking is a computational method used to predict the binding modes and interaction strengths between proteins and small molecules. In this study, molecular docking was performed to investigate the interactions between ten active compounds of WT and two core targets, and their docking scores and binding modes were evaluated. It is generally accepted that a docking score of less than 0 kcal/mol indicates that the active compound can spontaneously bind to the target, a docking score below −5 kcal/mol suggests good binding activity, and a docking score below −7 kcal/mol indicates strong binding activity [30,31]. The results showed that the docking scores of the 10 active compounds of WT with the 2 core targets ranged from −5.3 kcal/mol to −8.7 kcal/mol (Supplementary Table S7). Among these, 10 combinations had docking scores between −5 kcal/mol and −7 kcal/mol, and 10 combinations had docking scores below −7 kcal/mol, indicating strong binding activity between the WT active compounds and the 2 core targets. The docking scores of the reference drug acarbose with CTSV and NOD1 were −7.6 kcal/mol and −6.2 kcal/mol, respectively. Four active compounds demonstrated higher docking scores with CTSV than acarbose, while 10 active compounds exhibited higher docking scores with NOD1. This suggests that these 10 compounds are key contributors to the anti-DM effect of WT.

Among them, theobromine improves non-alcoholic fatty liver in obese mice by regulating the mTOR signaling pathway, lowering blood glucose and lipid levels, and additionally reducing blood glucose by inhibiting *α*-glucosidase activity [31,32]. Theophylline enhances insulin secretion and improves hypoglycemia awareness [33]. It has been reported that theophylline protects pancreatic *β*-cells through its immunosuppressive effects [34]. Caffeine lowers blood glucose and improves insulin sensitivity by reducing the expression of inflammatory factors and improving fatty liver [35]. When used in combination with anti-DM drugs, caffeine enhances drug bioavailability and significantly lowers blood glucose, demonstrating a synergistic anti-DM effect [36]. In addition, GC helps reduce postprandial hyperglycemia by inhibiting the activities of *α*-amylase and *α*-glucosidase [37]. C lowers blood glucose and alleviates DM symptoms by improving insulin sensitivity, inhibiting *α*-glucosidase activity, and enhancing antioxidant capacity. Furthermore, it reduces oxidative stress, preventing DM complications [38,39,40]. EGCG exhibits significant anti-DM effects, including lowering blood glucose levels by improving insulin sensitivity, inhibiting hepatic gluconeogenesis, and inhibiting *α*-glucosidase [41,42,43]. GCG suppresses carbohydrate absorption, enhances insulin sensitivity, and possesses anti-glycation properties [37,44]. ECG reduces the risk of DM complications and postprandial hyperglycemia by inhibiting protein glycation and the activities of *α*-amylase and *α*-glucosidase [45]. GA protects pancreatic cells and reduces DM-induced oxidative stress and complications by improving glucose metabolism and enhancing antioxidant activity [46,47]. GA, when combined with conventional drugs such as metformin, enhances the hypoglycemic effect [48].

Upon ranking the docking scores, it was found that EGCG and ECG were the best ligands for CTSV, with docking scores of −8.7 kcal/mol. C was the best ligand for NOD1, with a docking score of −8 kcal/mol. EGCG formed six hydrogen bonds with CTSV residues ASP163 (2.33 Å), GLY7 (2.54 Å), HIS141 (2.37 Å), GLN145 (2.65 Å), THR52 (2.95 Å), and GLN53 (2.17 Å), a hydrophobic interaction with ARG9 (4.93 Å), an electrostatic interaction with ARG11 (4.14 Å), and repulsive forces with THR58 (2.98 Å and 1.16 Å) and ARG9 (1.46 Å) (Figure 3C). ECG formed eight hydrogen bonds with CTSV residues GLY7 (2.70 Å), THR52 (2.58 Å and 2.97 Å), HIS141 (2.35 Å), THR58 (1.98 Å), ARG9 (2.12 Å), LYS19 (2.57 Å), and GLN53 (2.28 Å), a hydrophobic interaction with ARG9 (4.85 Å), and electrostatic interactions with ARG11 (3.96 Å and 4.22 Å) (Figure 3D). C formed two hydrogen bonds with NOD1 residues THR66 (2.11 Å) and GLU56 (2.66 Å), a hydrophobic interaction with ALA55 (5.23 Å) and PHE4 (4.87 Å), and repulsive forces with ALA60 (1.07 Å) (Figure 3E). In CTSV, acarbose formed ten hydrogen bonds with the residues ASN161 (2.42 Å), LEU162 (2.24 Å and 1.99 Å), GLN145 (2.14 Å), GLY140 (2.10 Å), THR54 (2.25 Å), THR58 (2.00 Å), ARG11 (2.31 Å), ARG9 (2.00 Å), and GLY140 (3.22 Å), as well as a hydrophobic interaction with LEU4 (2.95 Å) (Figure 3F). In NOD1, acarbose established four hydrogen bonds with ARG35 (2.30 Å), ASN36 (2.32 Å and 2.03 Å), and GLN2 (3.06 Å), along with a hydrophobic interaction with CYS39 (4.25 Å) (Figure 3G). In comparison, the binding of EGCG and ECG to CTSV relied on multiple hydrogen bonds, hydrophobic interactions, and electrostatic forces, demonstrating superior binding affinity compared to acarbose. C exhibited the strongest binding affinity to NOD1, primarily through hydrogen bonds and hydrophobic interactions. Although acarbose formed several hydrogen bonds, its binding affinity to CTSV and NOD1 was relatively weaker due to insufficient hydrophobic and electrostatic interactions.

EGCG and ECG have demonstrated significant binding affinity to CTSV, highlighting their potential as lead compounds for the development of anti-diabetic therapies targeting this protease. Given that CTSV plays a crucial role in regulating pancreatic *β*-cell apoptosis, inhibiting CTSV may help preserve *β*-cell function and maintain islet integrity. Both EGCG and ECG interact with multiple CTSV residues, reinforcing their therapeutic potential. However, the catechol groups in EGCG and ECG are part of pan-assay interference compounds (PAINS), which are prone to generating false-positive results in high-throughput screenings, as noted by Baell and Holloway [49]. This raises concerns about the reliability of initial pharmacological assessments.

To ensure the validity of their therapeutic effects, future research should focus on improving the bioavailability and absorption of EGCG and ECG while rigorously validating their anti-diabetic activity to eliminate the risk of PAINS-related artifacts. Further experimental studies are essential to accurately evaluate the therapeutic potential of these compounds. Additionally, strategies such as chemical modification or advanced drug delivery systems, including nanoparticles, could be explored to enhance the bioavailability and efficacy of EGCG and ECG in diabetes treatment. A deeper understanding of their molecular interactions will provide a critical foundation for the development of diabetes treatments based on these natural compounds.

### 3.7. Molecular Simulation

**Figure 4:**
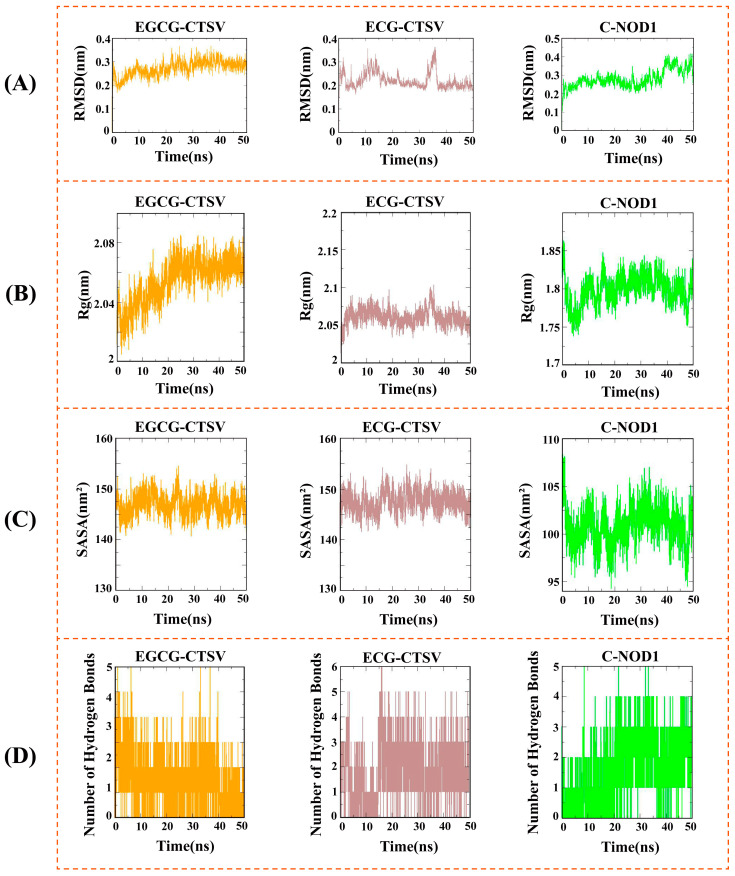
Molecular dynamics simulations of the three complexes were performed as follows: (**A**) Root Mean Square Deviation (RMSD) of the three complexes; (**B**) Radius of gyration (Rg) of the three complexes; (**C**) Solvent Accessible Surface Area (SASA) of the three complexes; and (**D**) Number of hydrogen bonds in the three complexes.

To further investigate the stability of protein-ligand interactions, molecular dynamics simulations were conducted on the EGCG-CTSV, ECG-CTSV, and C-NOD1 protein-ligand complexes. The root mean square deviation (RMSD) curve was used to reveal fluctuations in protein conformation, where RMSD values below 1 nm generally indicate the relative stability of protein-ligand interactions under physiological conditions [50]. The results showed that the RMSD of EGCG-CTSV, ECG-CTSV, and C-NOD1 fluctuated within 0.27 ± 0.03 Å, 0.22 ± 0.03 Å, and 0.28 ± 0.05 Å, respectively, suggesting that these complexes formed relatively tight interactions during binding (Figure 4A). The radius of gyration (Rg) was used to assess the structural stability of the protein-ligand complexes [51]. The Rg values for the three complexes were 2.05 ± 0.02 nm, 2.06 ± 0.01 nm, and 1.80 ± 0.02 nm, respectively, indicating that the complexes adopted a more compact and stable structure (Figure 4B). Solvent-accessible surface area (SASA) was employed to provide insights into protein folding and hydrophobicity [52]. The SASA values of the three complexes remained relatively stable at 146.85 ± 2.07 nm^2^, 147.56 ± 2.04 nm^2^, and 100.81 ± 1.97 nm^2^, respectively, indicating that no significant structural changes occurred during the simulations (Figure 4C). The number of hydrogen bonds reflected the strength of protein-ligand binding [53], and as shown in Figure 4D, the hydrogen bond density and strength for EGCG-CTSV, ECG-CTSV, and C-NOD1 were consistently high.

To further explore the stability of protein-ligand interactions, MM/GBSA and MM/PBSA methods were used to evaluate the binding free energy of the three complexes. Both methods are widely applied to assess the binding potential of protein complexes. The MM/GBSA results (Table 2) indicated that the binding free energy of EGCG-CTSV, ECG-CTSV, and C-NOD1 was −25.48 kcal/mol, −27.52 kcal/mol, and −27.52 kcal/mol, respectively. The binding free energy is composed of van der Waals energy, electrostatic energy, solvation energy, and solvent-accessible surface area energy. The van der Waals energy for the three complexes was −44.67 kcal/mol, −40.04 kcal/mol, and −36.84 kcal/mol, respectively; the electrostatic energy was −4.80 kcal/mol, −7.5 kcal/mol, and −7.13 kcal/mol, respectively; the solvation energy was 28.78 kcal/mol, 24.78 kcal/mol, and 20.62 kcal/mol, respectively; and the solvent-accessible surface area energy was −4.78 kcal/mol, −4.71 kcal/mol, and −4.71 kcal/mol, respectively. These results suggest that van der Waals energy, electrostatic energy, and solvent-accessible surface area energy contributed to complex binding, while solvation energy hindered complex binding. The MM/PBSA results (Table 3) were similar to those of MM/GBSA, with ECG-CTSV showing the highest binding free energy, followed by C-NOD1 and EGCG-CTSV. The binding free energy for the three complexes was −26.93 kcal/mol, −25.39 kcal/mol, and −23.29 kcal/mol, respectively.

In conclusion, molecular dynamics simulations revealed that the EGCG-CTSV, ECG-CTSV, and C-NOD1 complexes exhibited stable binding, characterized by favorable contributions from van der Waals, electrostatic, solvation, and solvent-accessible surface area energies.

## 4. Conclusions

This study employed a multi-faceted approach to systematically investigate the anti-DM active compounds in WT and their potential mechanisms. This approach included polyphenol-targeted metabolomics, in vitro antioxidant and *α*-glucosidase activity assays, network pharmacology, GEO data analysis, molecular docking, and molecular dynamics simulations. These findings, combined with antioxidant and *α*-glucosidase activity assays, identified 10 key active compounds in WT that exhibit significant anti-DM activity. Additionally, network pharmacology, GEO data analysis, and ROC analysis highlighted CTSV and NOD1 as critical targets of these compounds, implicating pathways related to signal transduction, apoptosis, and immune response in their anti-DM effects. Crucially, molecular docking revealed that these active compounds bind to the core targets via hydrogen bonding and hydrophobic interactions. Molecular dynamics simulations further supported the stability of these protein–ligand interactions.

The previous research has demonstrated that WT can regulate blood glucose levels and improve insulin sensitivity. This study systematically evaluated the antidiabetic potential of WT, identifying its key bioactive compounds, target proteins, and related mechanisms of action. These findings provide valuable insights for developing diabetes treatments based on natural products. Despite the significant discoveries revealed in this study, the limitations of computational simulations should be acknowledged. The complexity of biological systems makes it difficult for models to fully replicate all variables and interactions. Therefore, future research should aim to further validate and explore the mechanisms by which these 10 active compounds regulate diabetes-related pathways through in vitro and in vivo studies, as well as molecular biology approaches.

## Figures and Tables

**Table 1:** Results of antioxidant activity and α-glucosidase inhibition assays.

Samples	DPPH	ABTS	FRAP	HRSA	α-Glucosidase Activity
Control	1.31 ± 0.19 ^b^	1.27 ± 0.31 ^c^	0.13 ± 0.03 ^c^	0.63 ± 0.08 ^c^	163.13 ± 4.82 ^a^
acarbose	74.50 ± 0.93 ^a^	94.92 ± 0.44 ^a^	28.49 ± 0.15 ^a^	15.99 ± 0.02 ^a^	8.74 ± 0.12 ^b^
WT	71.50 ± 0.39 ^a^	85.43 ± 0.10 ^b^	9.46 ± 0.14 ^b^	6.57 ± 0.45 ^b^	14.09 ± 1.48 ^b^

Different letters indicate a significant difference at the *p* < 0.05 level.

**Table 2:** The binding energy of the ligand-receptor complex under the MM/GBSA method is expressed in kcal/mol.

Parameters	EGCG-CTSV	ECG-CTSV	C-NOD1
Delta Van Der Waals Energy	−44.67 ± 0.66	−40.04 ± 2.58	−36.84 ± 0.14
Delta Electrostatic Energy	−4.80 ± 2.41	−7.56 ± 1.55	−7.13 ± 2.04
Delta Generalized Born Energy	28.78 ± 0.21	24.78 ± 0.30	20.62 ± 0.53
Delta Solvent Accessible Surface Area Energy	−4.78 ± 0.02	−4.71 ± 0.23	−4.17 ± 0.15
Total Binding Energy	−25.48 ± 2.51	−27.52 ± 3.03	−27.52 ± 2.11

**Table 3:** The binding energy of the ligand-receptor complex under the MM/PBSA method is expressed in kcal/mol.

Parameters	EGCG-CTSV	ECG-CTSV	C-NOD1
Delta Van Der Waals Energy	−44.67 ± 0.66	−40.04 ± 2.58	−36.84 ± 0.14
Delta Electrostatic Energy	−4.80 ± 2.41	−7.56 ± 1.55	−7.13 ± 2.04
Delta Polar Solvation Energy	29.67 ± 0.72	24.08 ± 1.35	21.34 ± 0.63
Delta Non-Polar Solvation Energy	−3.51 ± 0.02	−3.42 ± 0.16	−2.76 ± 0.03
Total Binding Energy	−23.29 ± 2.60	−26.93 ± 3.30	−25.39 ± 2.14

## Data Availability

The original contributions presented in the study are included in the article/Supplementary Material, further inquiries can be directed to the corresponding author.

## Supplementary Materials

The following supporting information can be downloaded at: https://www.mdpi.com/article/10.3390/foods13213354/s1, Supplementary Figure S1. Targeted Metabolomics Profiling of Polyphenols in White Tea; Supplementary Figure S2. Boxplot of Standardized GSE76896 Dataset; Supplementary Table S1. Targeted Metabolomics Analysis of Polyphenol Content in White Tea; Supplementary Table S2. Correlation between Active Components of White Tea, Antioxidant Activity, and *α*-Glucosidase Activity; Supplementary Table S3. Target Proteins of 10 Active Components in White Tea; Supplementary Table S4. Sample Information for GSE76896; Supplementary Table S5. Differentially Expressed Genes Information; Supplementary Table S6. Intersection Analysis; Supplementary Table S7. Docking Scores of 10 Active Compounds from White Tea and Positive Control Drugs with Two Core Targets.
