# Supplementary material for: Profiling of Potential Anti-Diabetic Active Compounds in White Tea: An Integrated Study of Polyphenol-Targeted Metabolomics, Network Pharmacology, and Computer Simulation
Source: Foods. 2024 Oct 22;13(21):3354. doi: 10.3390/foods13213354 (PMC11545757; doi:10.3390/foods13213354)
Supplement: Supplementary file 1 [file foods-13-03354-s001.zip › Supplementary Figure.pdf]

Supplementary Materials for

Profiling of Potential Anti-Diabetic Active Compounds in White Tea: An Integrated Study of Polyphenol-Targeted Metabolomics, Network Pharmacology, and Computer Simulation

Weiwei Wu, Zhiqiang Zheng, Zhihui Wang, Chenxi Gao, Yilin Liang, Wen Zeng and Weijiang Sun *

College of Horticulture, Fujian Agriculture and Forestry University, Fuzhou 350002, China; wweiwei0601@163.com (W.W.); zzq000422@163.com (Z.Z.); wzh1246900265@163.com (Z.W.); gaochenxii@163.com (C.G.); lyl908213@163.com (Y.L.); wenzeng_t@163.com (W.Z.)

* Correspondence: 000q020007@fafu.edu.cn

Supplementary Figure Material

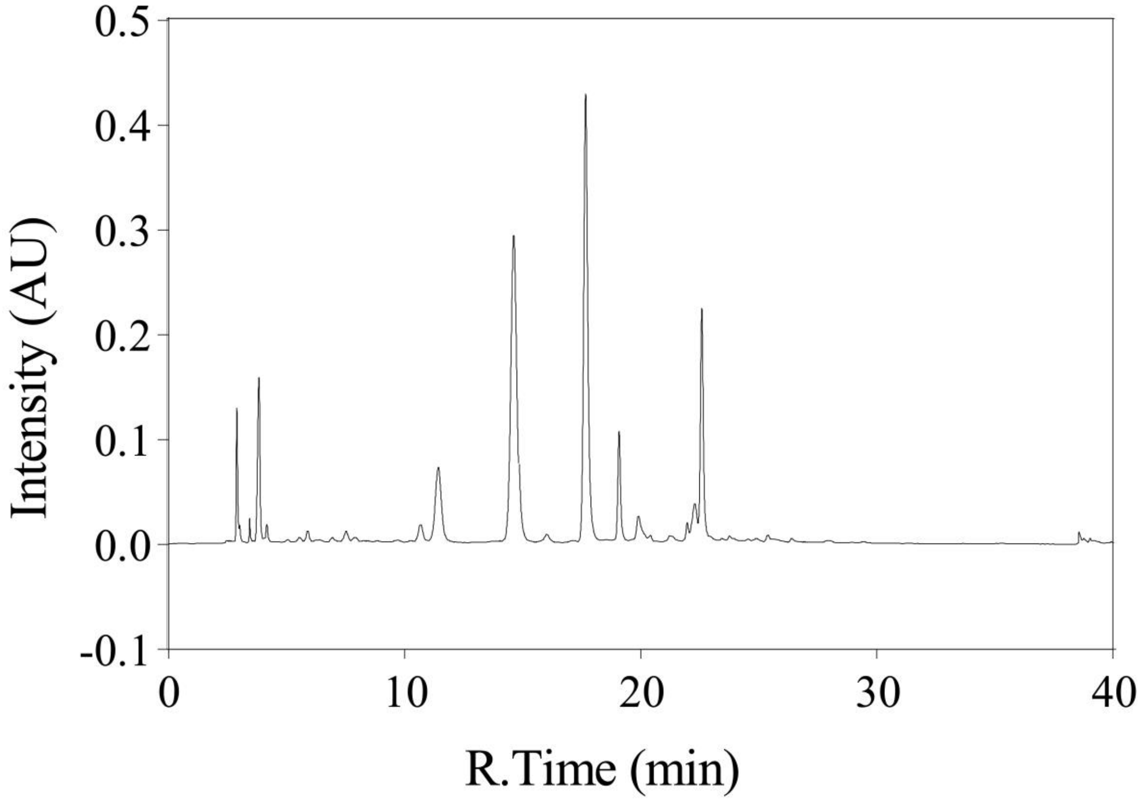

Supplementary Figure S1. Targeted Metabolomics Profiling of Polyphenols in White Tea.

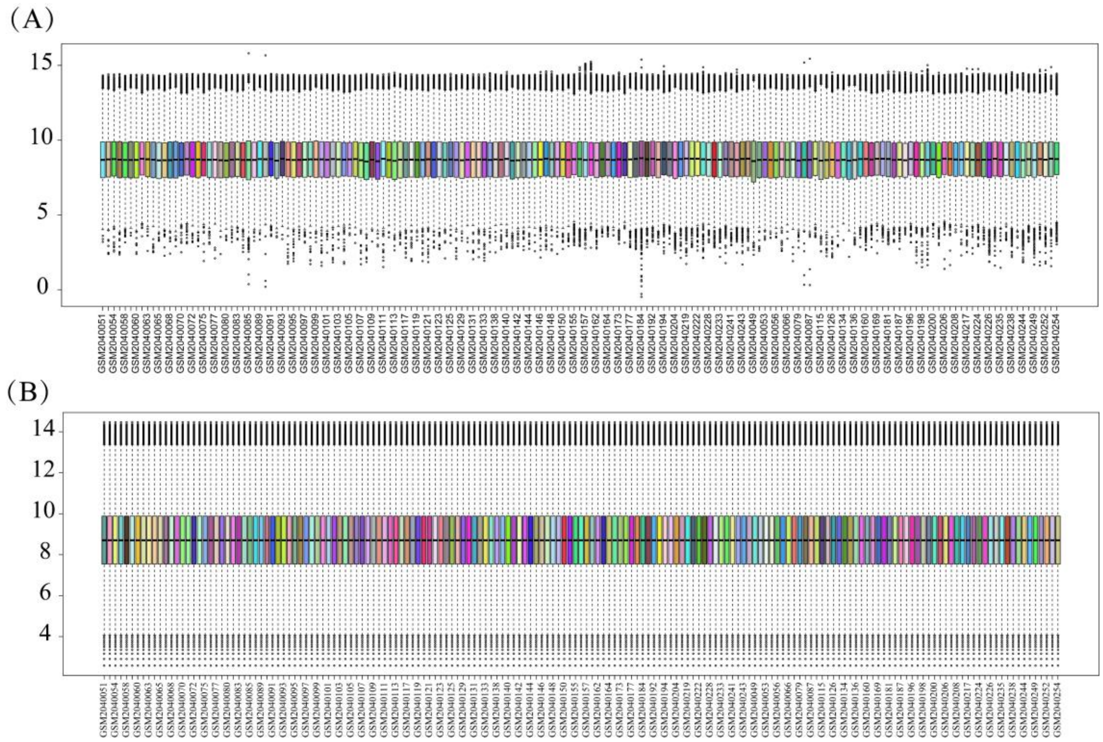

Supplementary Figure S2. Boxplot of Standardized GSE76896 Dataset.
